# Instantaneous Conversion of [^11^C]CO_2_ to [^11^C]CO via Fluoride‐Activated Disilane Species

**DOI:** 10.1002/chem.201701661

**Published:** 2017-05-17

**Authors:** Carlotta Taddei, Salvatore Bongarzone, Antony D. Gee

**Affiliations:** ^1^ Division of Imaging Sciences and Biomedical Engineering, King's College London, 4th Floor Lambeth Wing St. Thomas' Hospital London Lambeth Palace Road SE1 7EH United Kingdom

**Keywords:** carbon-11, carbonylation, isotopes, radiochemistry, silanes

## Abstract

The development of a fast and novel methodology to generate carbon‐11 carbon monoxide ([^11^C]CO) from cyclotron‐produced carbon‐11 carbon dioxide ([^11^C]CO_2_) mediated by a fluoride‐activated disilane species is described. This methodology allows up to 74 % conversion of [^11^C]CO_2_ to [^11^C]CO using commercially available reagents, readily available laboratory equipment and mild reaction conditions (room temperature). As proof of utility, radiochemically pure [carbonyl‐^11^C]*N*‐benzylbenzamide was successfully synthesized from produced [^11^C]CO in up to 74 % radiochemical yield (RCY) and >99 % radiochemical purity (RCP) in ≤10 min from end of [^11^C]CO_2_ delivery.

The short‐lived positron‐emitting radionuclide carbon‐11 (radioactive half‐life, 20.4 min) is generally produced in the form of [^11^C]CO_2_ by the ^14^N(p, α)^11^C nuclear reaction in the presence of trace amounts of oxygen (0.5–1 %). Due to the low chemical reactivity of CO_2_, only a limited number of methods have been developed to incorporate [^11^C]CO_2_ directly into functionalized molecules.^1^

For radiosynthetic applications, cyclotron‐produced [^11^C]CO_2_ is generally transformed into more reactive species, such as [^11^C]CH_3_I, [^11^C]COCl_2_, [^11^C]HCN and [^11^C]CO.^2^ Among these, [^11^C]CO can be used to produce a vast array of [carbonyl‐^11^C]‐containing molecules, for example [^11^C]ureas, [^11^C]amides, [^11^C]esters, [^11^C]carboxylic acids.1d, 3 These classes of compounds are of great interest as potential radiotracers for molecular imaging applications using positron emission tomography (PET) which allows the quantitative bio‐distribution and kinetics of the labelled compounds to be studied in vivo.^4^

[^11^C]CO is commonly produced by gas‐phase reduction of cyclotron‐produced [^11^C]CO_2_ on a metal surface (zinc or molybdenum) at high temperatures (400–800 °C).^5^ Although this method can produce [^11^C]CO in good yields (≈70 %), unless the catalyst is frequently replaced, the yields rapidly decrease over time and successive [^11^C]CO production cycles due to the oxidation of the metal surface.5b The method also requires dedicated and costly infrastructure.

Novel methodologies to reliably produce [^11^C]CO using a simple and readily available set‐up are therefore of high interest to enable the more widespread use of [^11^C]CO as a versatile starting material for carbon‐11 labelling applications.

An innovative [^11^C]CO_2_ to [^11^C]CO chemical conversion using [^11^C]silacarboxylic acids based on synthetic chemistry studies has been reported recently from our group and others.^6^ This methodology allows the production of [^11^C]CO by carboxylation of freshly prepared silyl lithium derivatives with [^11^C]CO_2_ with subsequent addition of tetra*‐n‐*butylammonium fluoride (TBAF) as an activator to trigger [^11^C]CO release. This represents a rapid and efficient methodology based on a simple set‐up. However, it requires time‐consuming preparation of the silyl lithium derivatives and the addition of a fluoride salt in stoichiometric excess to produce [^11^C]CO which somewhat detracts from the attractiveness of the approach for routine application.

Recently, disilanes have been reported to be useful sources of CO in synthetic chemistry using catalytic amounts of fluoride salts.^7^ Disilanes were therefore identified as new [^11^C]CO_2_ to [^11^C]CO converting agents to potentially overcome the remaining caveats associated with [^11^C]CO production using the existing [^11^C]silacarboxylic acids approach.6a, 6b

All RCYs and [^11^C]CO yields are reported as decay corrected values. A simple two‐vial set‐up (vial A and B) is used (Figure 1).6a Vial A contains a disilane species ((R_3_Si)_2_) and a fluoride salt dissolved in an aprotic solvent (e.g. THF, dioxane, DMSO). Vial B contains carbonylation reagents for the synthesis of [carbonyl‐^11^C]‐*N*‐benzylbenzamide ([^11^C]**3**, Scheme 1).^8^ The cyclotron‐produced [^11^C]CO_2_ is delivered directly into vial A in a stream of helium gas (Figure 1). An Ascarite trap is placed between vials A and B to trap any unreacted [^11^C]CO_2_. The produced [^11^C]CO is consumed by the carbonylation reaction in vial B giving [^11^C]**3** (Figure 1). The [^11^C]CO percent yield is calculated as the radioactivity trapped in vial B divided by the sum of the total radioactivity measured in vial A, vial B and the Ascarite trap at end of [^11^C]CO production. The crude reaction mixture of vial B is analyzed by radio‐HPLC to determine the RCP of [^11^C]**3** (Supporting Information Figure S1).

**Figure 1:**
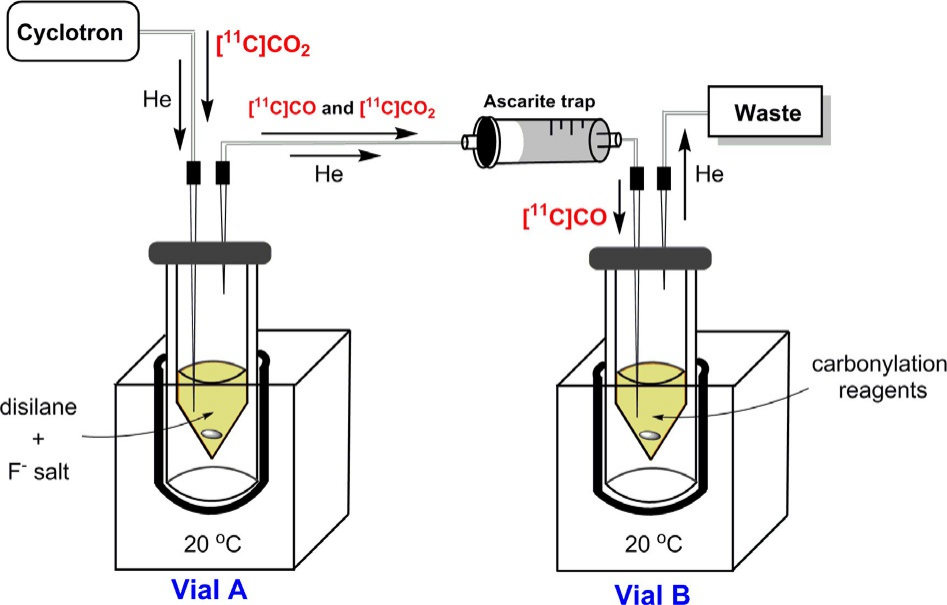
Two‐vial set‐up (vial A and vial B).

**Scheme 1:**
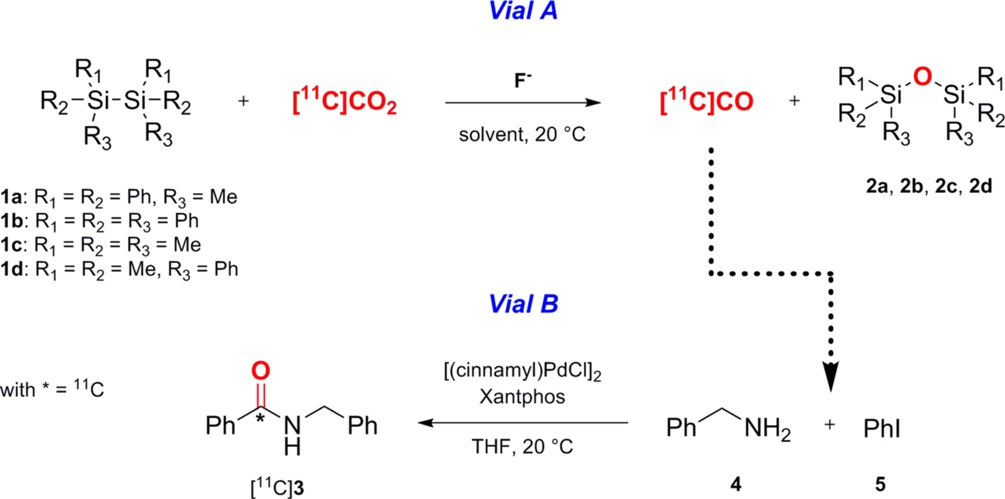
[^11^C]CO synthesis in vial A and ^11^C‐carbonylation reaction in vial B.

To confirm that the [^11^C]CO trapped in vial B gave accurate estimates of the process yields, the radioactivity of the waste line from vial B was time‐monitored. This showed constant values (<0.3 % of total radioactivity) from the end of [^11^C]CO_2_ delivery from the cyclotron until end of the [^11^C]CO carbonylation reaction in all experiments (Supporting Information Figure S2).

Initially we investigated disilane **1 a** (Scheme 1) using a variety of fluoride species, CsF,^9^ KF,^9^ KHF_2_ 9 and TBAF (entries 1–4, Table 1) in vial A. CsF and KF gave very low [^11^C]CO yields, (entries 1 and 2, Table 1). Whereas, KHF_2_ and TBAF gave [^11^C]CO in RCYs of 4 and 9 %, respectively (entries 3 and 4, Table 1). Oxygen‐based anion sources were not tested since synthetic chemistry studies have shown that these anions, such as acetate (e.g. KOAc) require higher temperatures for the CO_2_ to CO process to occur.^7^ Our aim was to develop a radiosynthesis method to convert [^11^C]CO_2_ to [^11^C]CO at room temperature. Due to the higher [^11^C]CO yield obtained with TBAF, we decided to use TBAF as the fluoride salt for subsequent experiments.

**Table 1:** Reaction conditions optimization.

Entry^[a]^	Activator	Equiv.	Solvent	[^11^C]CO yield [%]^[b]^	[^11^C]**3** RCP [%]^[c]^
1	CsF	0.2	THF	1	nd^[e]^
2	KF	0.2	THF	2	nd^[e]^
3	KHF_2_	0.2	THF	4	>99
4	TBAF	0.2	THF	9	>99
5	TBAF	0.2	dioxane	4	>99
6	TBAF	0.2	DMF	2	>99
7	TBAF	0.2	DMSO	1	>99
8	TBAF	0.2	DME	1	>99
9	TBAF	0.2	Et_2_O	0.5	90
10	TBAF	0.5	THF	1	90
11	TBAF	1.0	THF	1	>99
12	TBAF	2.0	THF	1	>99
13	TBAF	10.0	THF	1	>99
14^[d]^	TBAF	0.1	THF	32±2	>99
15	TBAF	0.05	THF	25	>99
16^[f]^	TBAF	0.1	THF	0	–
17^[g]^	–	–	THF	0	–
18^[h]^	–	–	THF	0	–
19	TBAB	0.1	THF	1	nd^[e]^
20	TBACl	0.1	THF	1	nd^[e]^

[a] All the experiments were performed with: vial A: **1 a** (63.5 mg, 0.161 mmol, 1.0 equiv), fluoride source and solvent (900 μL); vial B: **4** (50.24 μL, 0.46 mmol, 46.0 equiv), **5** (1.12 μL, 0.01 mmol, 1.0 equiv), [(cinnamyl)PdCl]_2_ (3.6 mg, 0.007 mmol, 0.07 equiv), Xantphos (4.0 mg, 0.007 mmol, 0.07 equiv) and THF (450 μL). [b] Calculated as a percentage by measurement of the radioactivity in vial B divided by the total radioactivity in the system at end of [^11^C]CO production. [c] RCP estimated from radio‐HPLC analysis of the crude reaction mixture of vial B. [d] Average of three experiments. [e] Radio‐HPLC analysis of vial B was not performed. [f] Absence of **1 a**. [g] Absence of TBAF and **1 a**. [h] Absence of TBAF.

In order to evaluate the influence of solvent on the reaction efficiency, a range of different aprotic solvents were screened (entries 4–9, Table 1). Aprotic solvents were chosen since these are reported to increase the solubility of disilane species and the reactivity of the fluoride anion in solution.^10^ With THF as a solvent, a [^11^C]CO yield of 9 % was obtained (entry 4, Table 1). Whereas, when dioxane and DMF were used, [^11^C]CO RCYs of 4 % and 2 % were achieved, respectively (entries 5 and 6, Table 1). Lower [^11^C]CO yields (≤1 %) were obtained in DMSO, DME and Et_2_O (entries 7–9, Table 1). Therefore, THF was chosen as the solvent to optimize the amount of TBAF.

Increasing the amount of TBAF from 0.2 to 10 equivalents resulted in a decrease of [^11^C]CO RCYs (entries 10–13 vs. entry 4, Table 1). Since higher amounts of TBAF did not provide yield improvements, we decided to decrease the equivalents of TBAF. Surprisingly, 0.1 equivalent of TBAF yielded the instantaneous production of [^11^C]CO in up to 32 % yield (entry 14, Table 1). By further decreasing the TBAF content to 0.05 equivalent, a [^11^C]CO yield of 25 % was achieved (entry 15, Table 1). Therefore, by reducing the amount of TBAF from 0.2 to 0.05 equivalents we observed a trend (Supporting Information Figure S6) which showed a maximum [^11^C]CO yield at 0.1 equivalents (32±2 %) and two lower values at 0.05 (25 %) and 0.2 equivalents (9 %) of TBAF. Additional optimization of TBAF equivalencies between these values were not explored as they were not anticipated to produce any further yield gains. These results indicated 0.1 equivalents TBAF as being optimum under these reaction conditions.

[^11^C]CO was not produced in the absence of TBAF, disilane or TBAF/disilane complex (entries 16–18, Table 1). It was concluded that the conversion of [^11^C]CO_2_ to [^11^C]CO requires both reagents (disilane and TBAF) for the reaction to proceed.

Experiments substituting fluoride sources with tetra*‐n‐*butylammonium bromide (TBAB) and tetra*‐n‐*butylammonium chloride (TBACl) produced [^11^C]CO yields of only 1 % (entries 19 and 20, Table 1). No other equivalents of these salts were investigated since we wanted to test the comparative equivalence corresponding to the optimized TBAF conditions (entry 14). This result confirmed the relevance of the fluoride anion in promoting the [^11^C]CO_2_ to [^11^C]CO conversion. Furthermore, the electronegativity trend of halogens (F>Cl>Br) and the bond energy of silicon with halogens (Si−F≫Si−Cl>Si−Br)^11^ support the greater activating power of TBAF on [^11^C]CO_2_ to [^11^C]CO conversion compared to the other tetrabutylammonium salts tested (TBACl and TBAB).

Subsequently, the influence of the [^11^C]CO_2_ flow delivery rate from the cyclotron to the reaction system was investigated (Table 2). The cyclotron‐produced [^11^C]CO_2_ was bubbled directly into vial A in a stream of helium with a flow rate of 60 mL min^−1^. Any unreacted [^11^C]CO_2_ was removed by the Ascarite trap prior vial B (Figure 1). This set‐up yielded a [^11^C]CO_2_ to [^11^C]CO conversions up to 32 % based on total cyclotron‐produced [^11^C]CO_2_ (entry 1, Table 2) within 3 minutes from end of cyclotron bombardment (EOB). However, up to 20 % of cyclotron‐produced [^11^C]CO_2_ was trapped in the Ascarite. It was suspected that this was due to the high flow rate used for the [^11^C]CO_2_ delivery into vial A.

**Table 2:** Optimized reaction conditions at different [^11^C]CO_2_ flow delivery rates.

Entry^[a]^	Flow rate [mL/min]	[^11^C]CO yield [%]^[b]^	[^11^C]**3** RCP [%]^[c]^
1 (*n*=3)	60	32±2	>99
2 (*n*=5)	10	59±6	>99
3 (*n*=2)	30	44±4	>99
4 (*n*=3)	5	57±6	>99

[a] All the experiments were performed with: Vial A: **1 a** (63.5 mg, 0.161 mmol, 1.0 equiv), TBAF (4.2 mg, 0.016 mmol, 0.1 equiv) and THF (900 μL); vial B: **4** (50.24 μL, 0.46 mmol, 46.0 equiv), **5** (1.12 μL, 0.01 mmol, 1.0 equiv), [(cinnamyl)PdCl]_2_ (3.6 mg, 0.007 mmol, 0.07 equiv), Xantphos (4.0 mg, 0.007 mmol, 0.07 equiv) and THF (450 μL). [b] Calculated as a percentage by measurement of the radioactivity in vial B divided by the total radioactivity in the system at end of [^11^C]CO production. [c] RCP estimated from radio‐HPLC analysis of the crude reaction mixture of vial B. *n*=number of experiments.

By decreasing the flow rate of [^11^C]CO_2_ delivery to 10 mL min^−1^ using a needle valve prior the [^11^C]CO_2_ delivery line to vial A (Supporting Information Figure S3), the amount of [^11^C]CO_2_ trapped in the Ascarite decreased and the [^11^C]CO_2_ to [^11^C]CO conversion increased to 59 %, (entry 2, Table 2) within 10 minutes from EOB.^12^ Flow delivery rates of 30 mL min^−1^ (entry 3, Table 2) and 5 mL min^−1^ (entry 4, Table 2) were also investigated. A [^11^C]CO yield of up to 44 % was achieved at 30 mL min^−1^; whereas at 5 mL min^−1^ no significant difference in [^11^C]CO RCY (57 %) was observed from those obtained with a flow rate of 10 mL min^−1^.

The optimized reaction conditions for **1 a** using a 10 mL min^−1^ flow rate (entry 1, Table 3) were tested with different disilane species (**1 b**–**1 d**, Scheme 1). **1 b** was difficult to dissolve in THF and gave very low [^11^C]CO yields (entry 2, Table 3). **1 c** produced yields up to 35 % (entry 3, Table 3). Whereas, **1 d** gave the highest [^11^C]CO RCYs (≥74 %) within the disilane species tested (entry 4, Table 3).

**Table 3:** Investigated disilane species.

Entry^[a]^	Disilane	[^11^C]CO yield [%]^[b]^	[^11^C]**3** RCP [%]^[c]^
1^[d]^	**1 a**	59±6	>99
2	**1 b**	3	>99
3	**1 c**	35	>99
4^[d]^	**1 d**	74±6	>99

[a] All the experiments were performed with: vial A: **1 a**‐**1 d** (0.161 mmol, 1.0 equiv), TBAF (4.2 mg, 0.016 mmol, 0.1 equiv) and THF (900 μL) with [^11^C]CO_2_ flow delivery rate of 10 mL/min; vial B: **4** (50.24 μL, 0.46 mmol, 46.0 equiv), **5** (1.12 μL, 0.01 mmol, 1.0 equiv), [(cinnamyl)PdCl]_2_ (3.6 mg, 0.007 mmol, 0.07 equiv), Xantphos (4.0 mg, 0.007 mmol, 0.07 equiv) and THF (450 μL). [b] Calculated as a percentage by measurement of the radioactivity in vial B divided by the total radioactivity in the system at end of [^11^C]CO production. [c] RCP estimated from radio‐HPLC analysis of the crude reaction mixture of vial B. [d] Average of five experiments.

Based on these results, we suggest two potential reaction mechanisms (Scheme 2, mechanisms A and B). Both routes start from a TBAF‐activated disilyl anion species (**I**), which is formed when a catalytic amount of TBAF is in solution with a disilane in an aprotic solvent (e.g. THF). Indeed, past studies have shown the production of fluoride‐activated disilyl anion species, such as **I**, upon reaction with TBAF in the presence of a disilane and an aprotic solvent.^7^, 10b According to mechanism A, cyclotron‐produced [^11^C]CO_2_ reacts with **I** to generate intermediate **II**. This unstable intermediary ^11^C‐labelled species may undergo internal rearrangement to yield a silyl fluoride (**III**) and a silanol tetra*‐n‐*butylammonium salt (**IV**) with release of [^11^C]CO. Subsequent nucleophilic attack of **IV** on a disilane molecule (which is present in large excess in vial A) generates a silyl tetra*‐n‐*butylammonium salt (**V**) and a disiloxane species (**VI**). On the other hand, mechanism B takes into account the equilibrium between **I** with **III** and **V**. In this case, **V** may couple with cyclotron‐produced [^11^C]CO_2_ to generate a ^11^C‐labelled carboxylated species **VII**. This ^11^C‐labelled species can undergo internal rearrangement in the presence of free TBAF (in blue, Scheme 2) to yield **IV** with release of [^11^C]CO. Subsequently **IV** may attack a disilane molecule in a similar manner to mechanism A, to produce **V** and **VI**. However, experiments in the presence of excess of TBAF gave no [^11^C]CO production (entries 10–13, Table 1). The displacement of [^11^C]CO_2_ from complex **I** under excess of fluoride hinders the formation of complex **II** (mechanism A) or species **VII** (mechanism B) and the subsequent [^11^C]CO production. Previous studies have shown that the [^11^C]silacarboxylate species **VII** release [^11^C]CO only in the presence of an excess of TBAF.6a, 6b Therefore, the [^11^C]CO production through mechanism B is less likely to happen under deficient TBAF concentrations. Furthermore, the stable [^11^C]silacarboxylated species **VII** is not observed by radio‐HPLC analysis (Figure S7 vs. Figure S8, Supporting Information). These results in conjunction with synthetic chemistry studies^6^, ^7^, 10b, 13 suggest that mechanism A is the most likely route for [^11^C]CO_2_ to [^11^C]CO conversion mediated by fluoride‐activated disilane species.

**Scheme 2:**
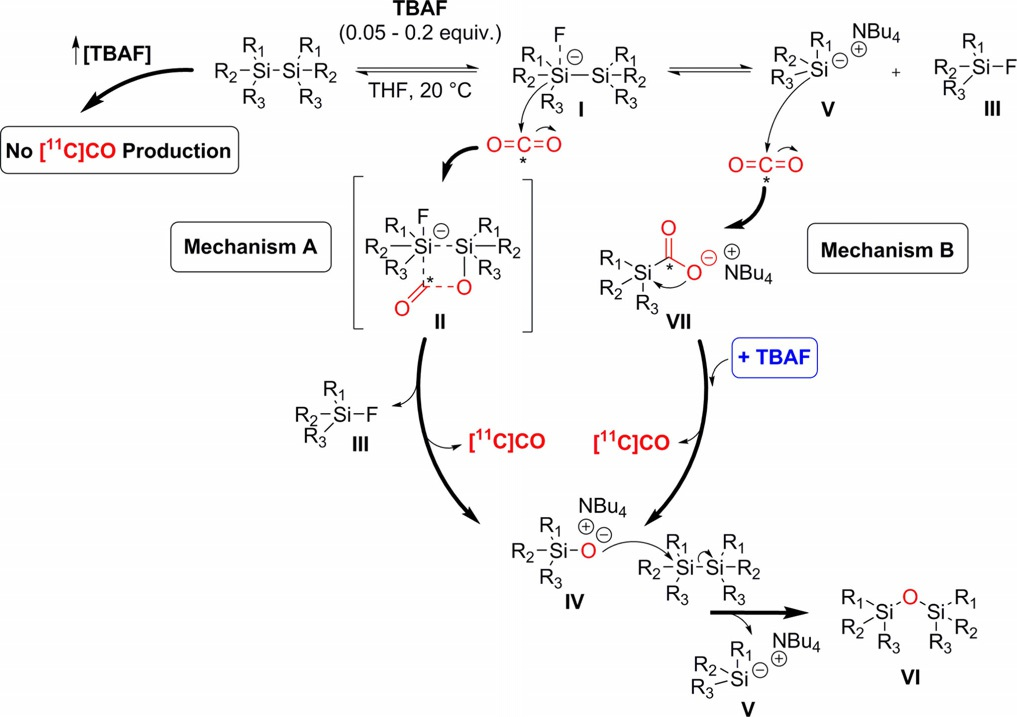
Proposed reaction mechanism.

A simple and rapid chemical conversion of [^11^C]CO_2_ to [^11^C]CO from disilane species in the presence of a catalytic amount of TBAF has been successfully developed. Up to 74 % of cyclotron‐produced [^11^C]CO_2_ was converted to [^11^C]CO within 10 minutes from end of [^11^C]CO_2_ delivery and under mild reaction conditions (room temperature).

This methodology is based on a simple laboratory set‐up and readily available reagents and is the first reported application of disilanes as [^11^C]CO releasing agents.

The produced [^11^C]CO was used in a model carbonylation reaction to yield [^11^C]**3** in up to 74 % RCY, >99 % RCP and short synthesis time (≤10 min from EOB).^14^

Due to the similar chemical behavior between disilanes and digermyl compounds observed in past studies,^15^ we predict that this latter class of reagents might be able to convert [^11^C]CO_2_ to [^11^C]CO in a similar manner to disilane species. Whereas past work has shown that the structurally related diboron species could not be activated by fluoride sources.^7^

In conclusion, this novel [^11^C]CO_2_ to [^11^C]CO approach has the potential to increase the utilization of [^11^C]CO in cyclotron‐based radiochemistry laboratories enhancing the prospects for development of new carbon‐11 labelled tracers for in vitro and in vivo PET imaging studies.

## Conflict of interest

The authors declare no conflict of interest.

## Supporting information

As a service to our authors and readers, this journal provides supporting information supplied by the authors. Such materials are peer reviewed and may be re‐organized for online delivery, but are not copy‐edited or typeset. Technical support issues arising from supporting information (other than missing files) should be addressed to the authors.

Supplementary
