# Supplementary material for: Instantaneous Conversion of [11C]CO2 to [11C]CO via Fluoride‐Activated Disilane Species
Source: Chemistry. 2017 May 17;23(32):7682–5. doi: 10.1002/chem.201701661 (PMC5488231; doi:10.1002/chem.201701661)
Supplement: Supplementary file 1 — Supplementary [file CHEM-23-7682-s001.pdf]

CHEMISTRY
A European Journal

Supporting Information

Instantaneous Conversion of [^{11}C]CO_2 to [^{11}C]CO via Fluoride-Activated Disilane Species

Carlotta Taddei, Salvatore Bongarzone, and Antony D. Gee*[a]

chem_201701661_sm_miscellaneous_information.pdf

Supporting Information
©Wiley-VCH 2016
69451 Weinheim, Germany

Instantaneous Conversion of [^{11}C]CO_2 to [^{11}C]CO *via* Fluoride-Activated Disilane Species

Carlotta Taddei, Salvatore Bongarzone, Antony D. Gee*

Abstract: The development of a fast and novel methodology to generate carbon-11 carbon monoxide ([^{11}C]CO) from cyclotron-produced carbon-11 carbon dioxide ([^{11}C]CO_2) mediated by fluoride-activated disilane species is described. This methodology allows up to 74% conversion of [^{11}C]CO_2 to [^{11}C]CO using commercially available reagents, readily available labware and mild reaction conditions (room temperature). As proof of utility, radiochemically pure [carbonyl-^{11}C]*N*-benzylbenzamide was successfully synthesized from produced [^{11}C]CO in up to 74% radiochemical yield (RCY) and > 99% radiochemical purity (RCP) in ≤ 10 min from end of [^{11}C]CO_2 delivery.

DOI: 10.1002/anie.2016XXXXX

WILEY-VCH

Table of Contents

General Methods and Materials......S2
HPLC Method......S2
Two-vial Set-up......S2
Representative procedure for [^{11}C]CO production......S3
Radio-HPLC of [^{11}C]*N*-benzylbenzamide and reference compound......S3
Radioactivity time-monitoring of Vial B and waste line......S4
Representative procedure with reduced [^{11}C]CO_2 delivery rate......S4
Two-vial set-up with reduced [^{11}C]CO_2 delivery rate......S4
Synthesis and characterization of disiloxane 2a......S5
TBAF equivalencies investigated in Table 1......S7
Radio-HPLC of Vial A at end of [^{11}C]CO production......S8
References......S8

General Methods and Materials

All chemicals and dry solvents were purchased from Sigma-Aldrich, Alfa Aesar and Santa Cruz Biotechnology and used as received. HPLC analysis was performed on an Agilent 1200 system equipped with a UV detector (λ=254 nm) and a β^+-flow detector coupled in series. ^{1}H and ^{13}C-NMR spectra were obtained using a BRUKER AVANCE DRX 400 MHz spectrometer. Gas chromatography-mass spectroscopy was performed using Agilent 7890B GC System (Column Agilent HP-5, length 30 m, diameter 0.32 mm, Film thickness 0.25 µm) combined with an Agilent 7200 Accurate-Mass Q-TOF GC/MS.[1] Elemental analysis was performed at the Science Centre of London Metropolitan University, London (UK). An Eckert and Ziegler Modular-Lab System equipped with magnetic stirring was used for the two-vial set-up needed in the carbon-11 experiments.

HPLC Method

A reverse-phase column (Agilent Eclipse XDB-C18, 4.6x150 mm) was used with a flow rate of 1 mL/min. The gradient was linear between 10–90% CH_3CN over 5 min (CH_3CN:H_2O, 10:90), isocratic between 5–9 min (CH_3CN:H_2O, 90:10), linear in 90–10% CH_3CN over 1 min and isocratic between 10–13 min (CH_3CN:H_2O, 10:90).

Two-vial Set-up

Two oven-dried vials (KX Microwave Vials, 2–5 mL, Vial A and KX Microwave Vials, 0.5–2 mL, Vial B) and crimp caps (Fisherbrand, centre hole with 3.0 mm PTFE seal aluminum silver 20 mm, part # 10132712) were used. All the lines used to permit the flow of gases were PTFE tubing (length: 10–20 cm, O.D.: 0.79 x 0.4 in., I.D.: 1/32 x 0.16 in.). Vial A and Vial B were placed in two heating blocks of an Eckert and Ziegler Modular-Lab. A P_2O_5 trap and one-way valve (BRAUN, normally closed backcheck valve, part # 415062) were placed before Vial A. An ascarite® trap consisting of a cartridge (Biosys Solutions Ltd, Fritted Empty MiniSpeed Cartridges, part # 2447) filled with ascarite (Sigma-Aldrich, 1310-73-2) was placed between Vial A and Vial B to trap unreacted [^{11}C]CO_2. Its presence was important to avoid unreacted [^{11}C]CO_2 to flow in Vial B and to lead us in quantifying the total [^{11}C]CO yield in Vial B. A waste bag was placed at the outlet of Vial B to avoid any escape of radioactive gases. Note: cyclotron-produced [^{11}C]CO_2 does not lead to side products in the carbonylation mixture used for the synthesis of [^{11}C]*N*-benzylbenzamide described in the manuscript.

WILEY-VCH

Representative procedure for [^{11}C]CO production

A disilane species (0.161 mmol, 1.0 equiv.), a fluoride source (0.05–2.0 equiv.) and a stirrer bar (Scientific Laboratory Supplies, STI4240, Stirrer Bar Oval PTFE 5x10 mm) were placed in a nitrogen-flushed dried 2–5 mL microwave vial. The reaction vial, Vial A, was then sealed and dry solvent (900 µL) was added under nitrogen atmosphere and magnetic stirring. Formation of a disilane fluoride-activated species was observed from the color change of the solution in Vial A from colorless to pale yellow. After a few minutes of stirring (1–5 min) at 20 °C, the cyclotron-produced [^{11}C]CO_2 was delivered to Vial A in a stream of helium with flow rate of ~ 60 mL/min. A cyclotron beam current of 5 µA was maintained for a bombardment time of 1 min for all reaction optimization experiments. The delivered [^{11}C]CO_2 was instantaneously converted to [^{11}C]CO by passage through the disilane fluoride-activated solution in Vial A. The produced [^{11}C]CO was transferred by helium purge to a second vial, Vial B, containing benzylamine (50.24 µL, 0.46 mmol, 46.0 equiv.), iodobenzene (1.12 µL, 0.01 mmol, 1.0 equiv.), [(cinnamyl)PdCl]$_2$ (3.6 mg, 0.007 mmol, 0.07 equiv.), Xantphos (4.0 mg, 0.007 mmol, 0.07 equiv.) and THF (450 µL). After 3 min from EOB, the transfer of the produced [^{11}C]CO to Vial B and the carbonylation reaction were finished. An aliquot of the crude reaction mixture of Vial B was quenched with mobile phase and analyzed by radio-HPLC to determine the RCP of [^{11}C]*N*-benzylbenzamide, [^{11}C]**3**. The cyclotron-produced [^{11}C]CO_2 was delivered and converted to [^{11}C]CO in up to 32% RCY within 3 min from EOB at this flow rate (60 mL/min). Note: the disilane species does not trap cyclotron-produced [^{11}C]CO_2 in the absence of TBAF. The presence of both reagents (TBAF and disilane) is necessary for the [^{11}C]CO_2 to [^{11}C]CO conversion to proceed.

Radio-HPLC of [^{11}C]*N*-benzylbenzamide and reference compound

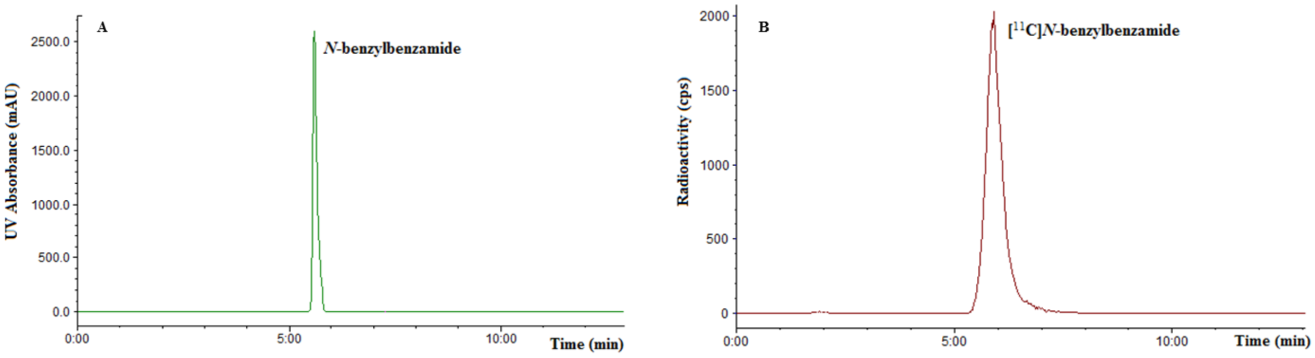

Figure S1: (A) UV-chromatogram *N*-benzylbenzamide (elution at 5.3 min). (B) Radio-chromatogram of Vial B ([^{11}C]*N*-benzylbenzamide eluting at 5.5 min). The delay between UV detector and radio-detector at flow rate 1 mL/min is 0.1–0.2 min.

WILEY-VCH

Radioactivity time-monitoring of Vial B and waste line

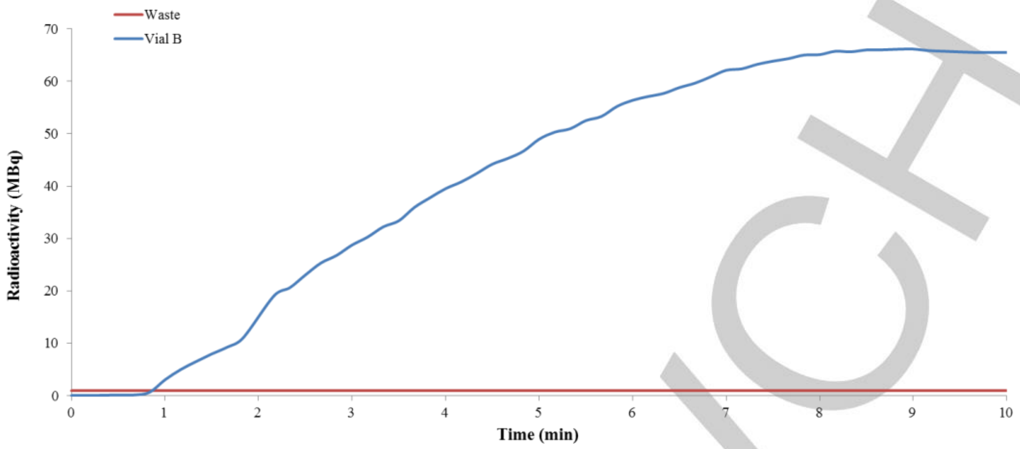

Figure S2: Radioactivity time-monitoring of Vial B and waste line from EOB (t_0).

Representative procedure with reduced [^{11}C]CO_2 delivery rate

Vial A and Vial B were prepared as described before (see “Representative procedure for [^{11}C]CO production”). A needle valve was placed between the delivery line of [^{11}C]CO_2 from the cyclotron and Vial A (Figure S3). The flow rate of helium was reduced to 10 mL/min and checked with a flow meter (FlowMark^{TM}, PerkinElmer, N9307086). [^{11}C]CO_2 was delivered and reacted in Vial A under helium purge (10 mL/min) for an additional 10 min after EOB. During this time, cyclotron-produced [^{11}C]CO_2 was converted to [^{11}C]CO and transferred to Vial B in up to 74% RCY within 10 min from EOB.

Two-vial set-up with reduced [^{11}C]CO_2 delivery rate

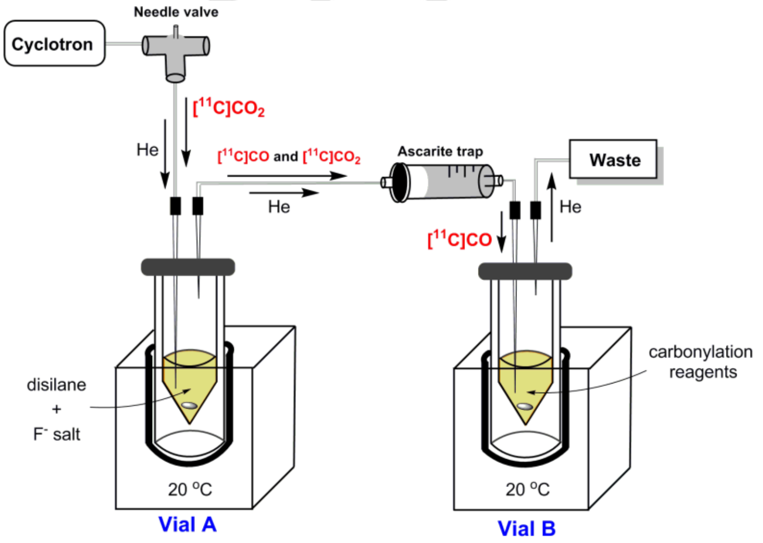

Figure S3: Two-vial set-up with needle valve prior Vial A for low flow delivery rate of [^{11}C]CO_2.

Synthesis and characterization of disiloxane 2a

Disilane **1a** (50 mg, 0.127 mmol, 1.0 equiv.), TBAF • xH_2O (3.3 mg, 0.0127 mmol, 0.1 equiv.) and a magnetic stirring bar were placed in a dried nitrogen-flushed microwave vial (2–5 mL). The vial was sealed under nitrogen and anhydrous THF (1 mL) was added under magnetic stirring. After the color of the solution had changed from clear colourless to clear pale yellow, the nitrogen atmosphere was exchanged with CO_2. Subsequently the solution was left to stir at room temperature (rt) for additional 3 hours under CO_2 atmosphere (balloon). After this period the reaction solution apparead clear and colourless indicating consumption of the disilane fluoride-activated species. Subsequently, the reaction vial was generosly flushed with nitrogen to eliminate any CO gas produced. The solvent was concentrated under vacuum and an aliquot of the crude reaction mixture was analysed by GC-MS(EI).[1] The starting material **1a**, product **2a**, silanol species **3a** and silyl fluoride **4a** were detected (Scheme S1). This confirmed the presence of the proposed final silane species involved in the [^{11}C]CO_2 to [^{11}C]CO mechanisms discussed in the manuscript. Following, the crude product was purified by flash silica column chromatography (95:5, hexane:EtOAc). The collected fractions containinig **2a** were concentrated and analyzed by NMR. Elemental analysis of **2a** is reported in Table S1.
Isolated yield of 2a: 37%.

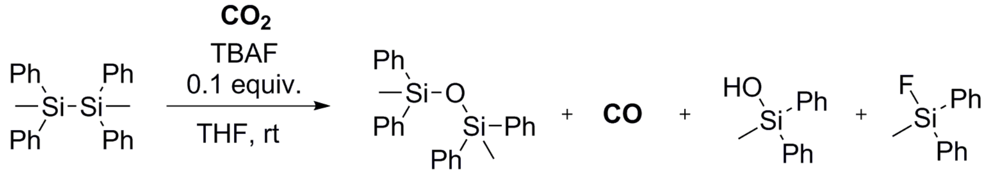

1a **2a** **3a** **4a**

Scheme S1: Synthesis of disiloxane **2a** and production of CO from disilane **1a**.

GC-MS(EI):

- **1a**: R_f = 14.491 min, found m/z = 394.16 [**1a**, $C_{26}H_{26}Si_2$]$^+$, calculated m/z = 394.00 [$C_{26}H_{26}Si_2$]$^+$
- **2a**: R_f = 13.955 min, found m/z = 395.13 [**2a**, $C_{25}H_{23}OSi_2$]$^+$, calculated m/z = 395.00 [$C_{25}H_{23}OSi_2$]$^+$
- **3a**: R_f = 9.916 min, found m/z = 199.06 [**3a**, $C_{12}H_{11}OSi$]$^+$, calculated m/z = 199.00 [$C_{12}H_{11}OSi$]$^+$
- **4a**: R_f = 8.986 min, found m/z = 201.05 [**4a**, $C_{12}H_{10}FSi$]$^+$, calculated m/z = 201.00 [$C_{12}H_{10}FSi$]$^+$

^{1}H NMR (400 MHz, $CDCl_3$, ppm): δ = 7.52 ppm (dd, *J*=7.9 Hz, 1.3 Hz, 8H), 7.40 – 7.26 ppm (m, 12H), 0.57 ppm (s, 6H).
^{13}C{^{1}H } NMR (101 MHz, $CDCl_3$, ppm): δ = 137.57 ppm, 133.99 ppm, 129.57 ppm, 127.72 ppm, -0.01 ppm.

^{1}H NMR of 2a

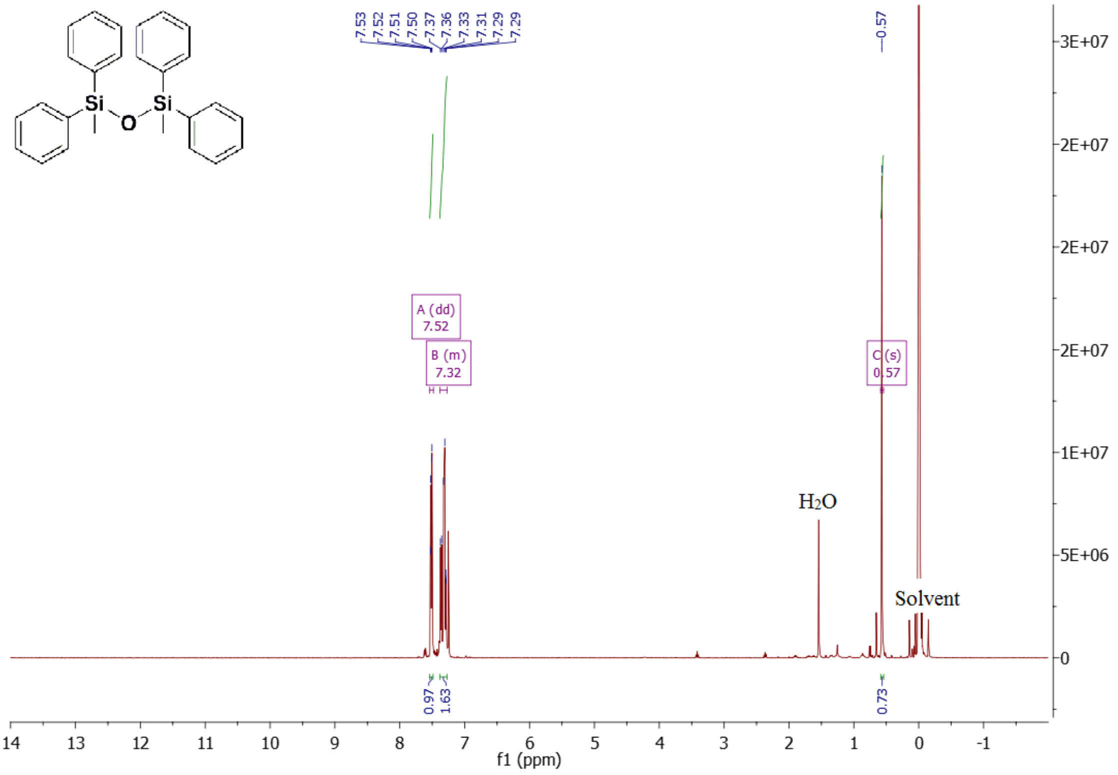

Figure S4:^{1}H NMR of **2a** in $CDCl_3$.

^{13}C{^{1}H} NMR of 2a

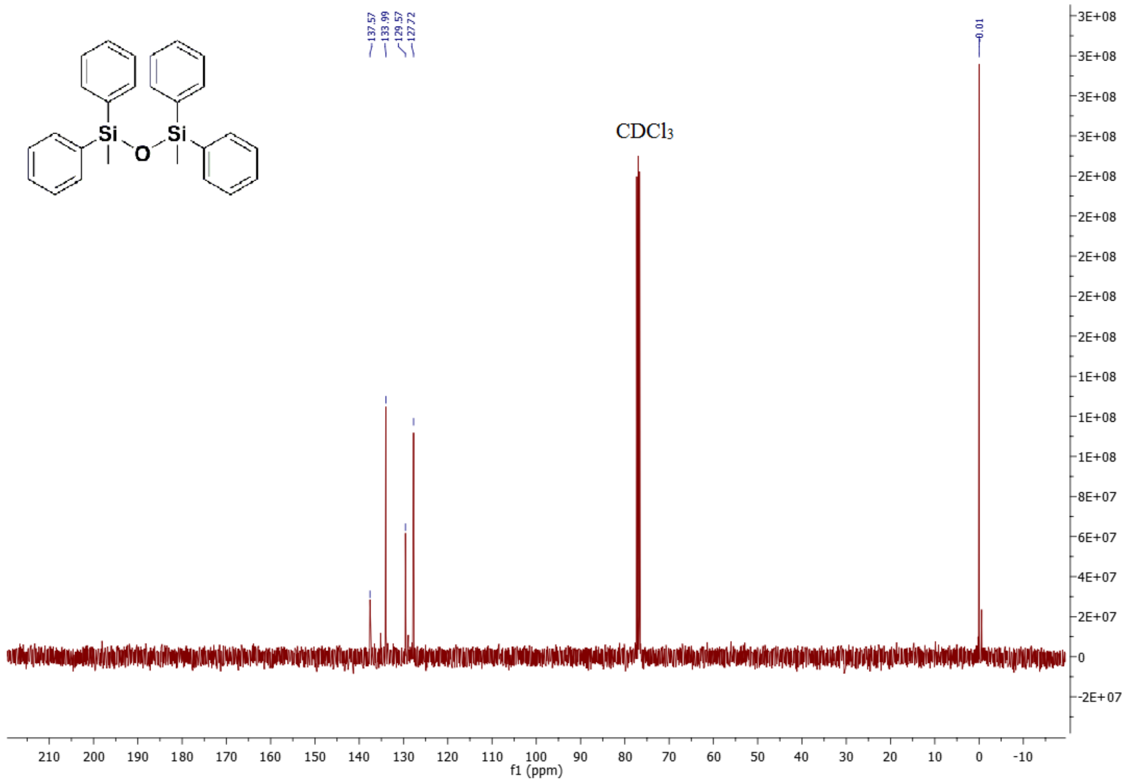

Figure S5: ^{13}C{^{1}H} NMR of **2a** in $CDCl_3$.

Elemental analysis of isolated disiloxane 2a

Table S1: Elemental analysis of **2a**.

Sample	%C	%H
**Experimental 1**	76.39	6.50
**Experimental 2**	76.29	6.57
**Theoretical**	76.04	6.38

TBAF equivalencies investigated in Table 1

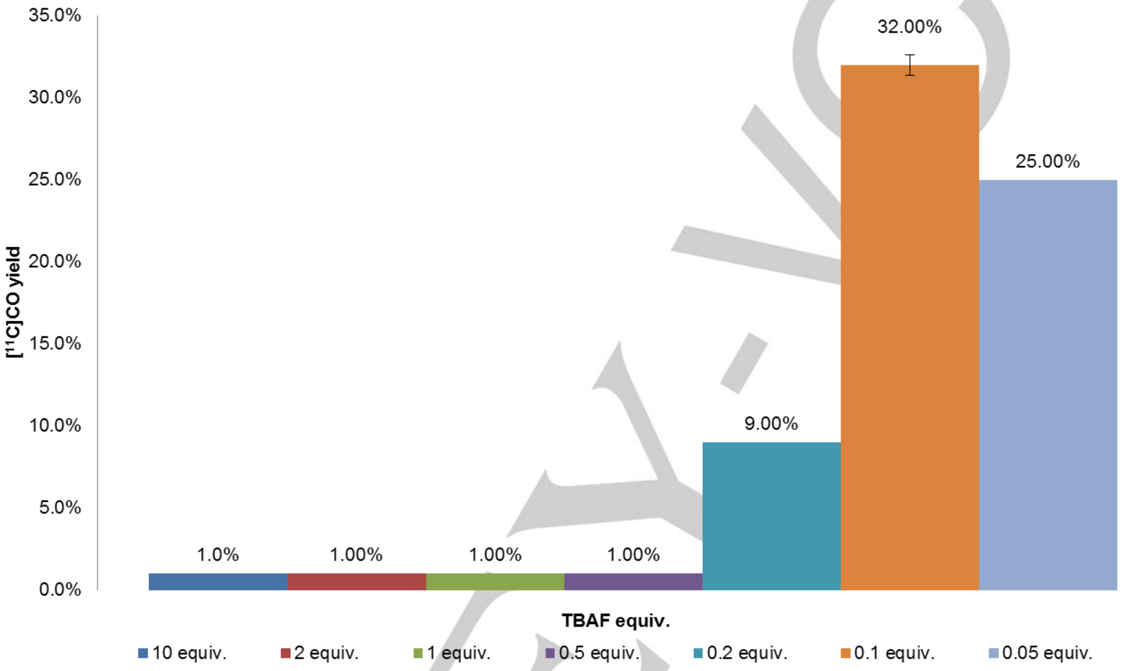

Figure S6: [^{11}C]CO yield for the TBAF equivalencies tested in Table 1.

WILEY-VCH

Radio-HPLC of Vial A at end of [^{11}C]CO production

At end of [^{11}C]CO_2 delivery and [^{11}C]CO production (Table 3, Entry 4) an aliquot of crude mixture of Vial A was quenched with mobile phase (CH_3CN:H_2O, 10:90) and analyzed by radio-HPLC. Note: from the HPLC analysis of the structurally related $Ph_2CH_3SiCOOH$ (Figure S8), used in our previous work[2], [^{11}C]silacarboxylated species were expected to elute at 7–8 min. No ^{11}C-labelled species was observed within this range (Figure S7) suggesting the absence of the corresponding [^{11}C]silacarboxylated derivative. Only two minor unknown ^{11}C-labelled species were detected within the initial 4 min of analysis.

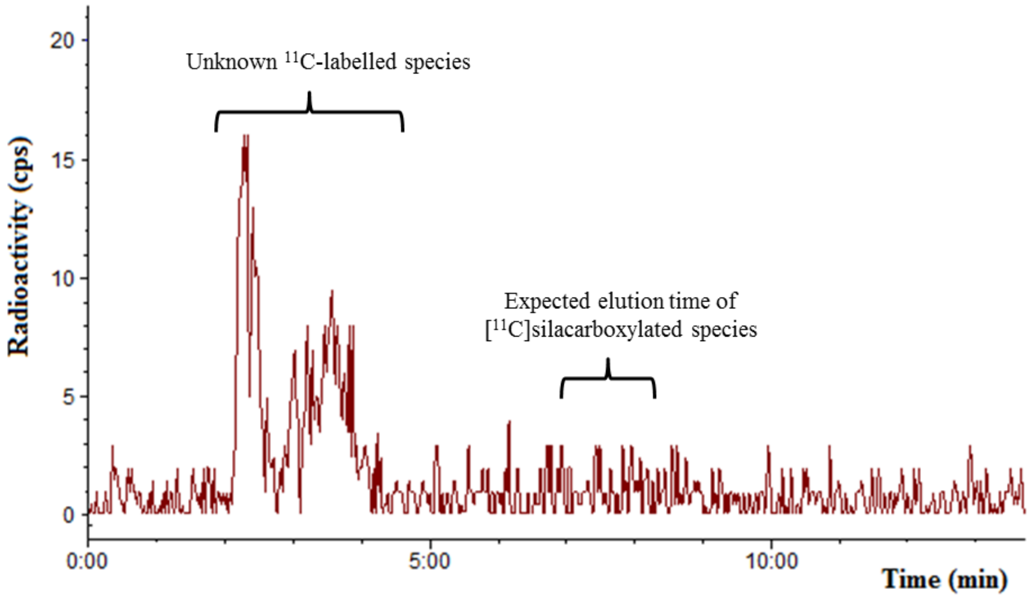

Figure S7: Representative radio-HPLC chromatogram of the crude mixture of Vial A (Table 1, Entry 4) after end of [^{11}C]CO production ("HPLC Method").

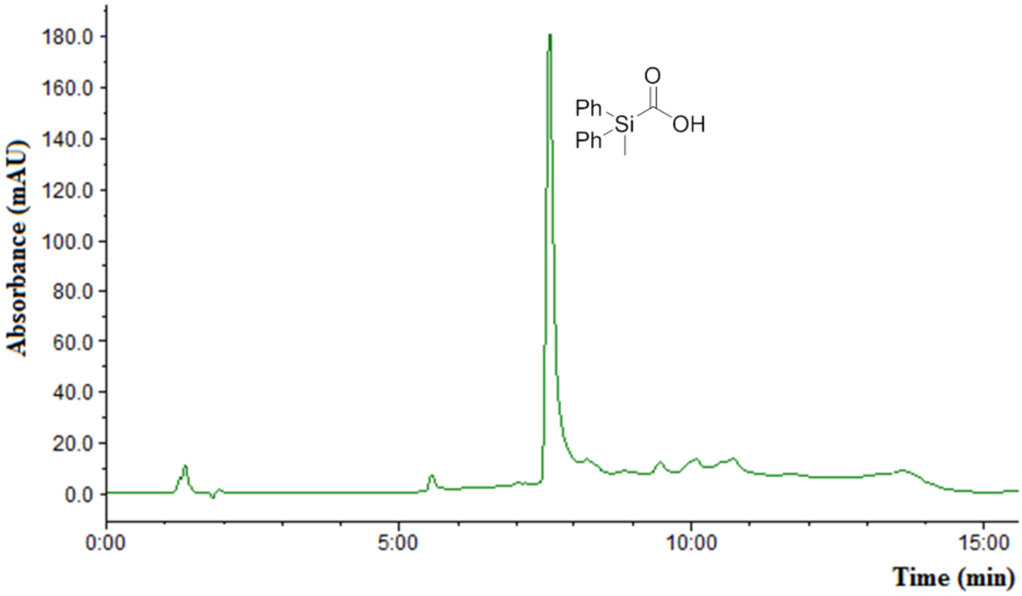

Figure S8: Representative HPLC chromatogram of $Ph_2CH_3SiCOOH$, eluting at 7.3 min ("HPLC Method").

References

[1] GC-MS(EI) analysis were carried out at the Chemistry Research Laboratory, Department of Chemistry, University of Oxford, 12 Mansfield Road, Oxford OX11 13TA, UK.

[2] C. Taddei, S. Bongarzone, A. K. Haji Dheere, A. D. Gee, *Chem. Commun.* **2015**, *51*, 11795-11797.

WILEY-VCH
